# Effect of Layer Directions on Internal Structures and Tensile Properties of 17-4PH Stainless Steel Parts Fabricated by Fused Deposition of Metals

**DOI:** 10.3390/ma14020243

**Published:** 2021-01-06

**Authors:** Yoshifumi Abe, Takashi Kurose, Marcelo V. A. Santos, Yota Kanaya, Akira Ishigami, Shigeo Tanaka, Hiroshi Ito

**Affiliations:** 1 Department of Organic Materials Science, Graduate School of Organic Materials Science, Yamagata University, 4-3-16 Jonan, Yonezawa, Yamagata 992-8510, Japan; tcs89036@st.yamagata-u.ac.jp (Y.A.); vergne_marcelo@taisei-kogyo-net.co.jp (M.V.A.S.); akira.ishigami@yz.yamagata-u.ac.jp (A.I.); 2 Research Center for GREEN Materials & Advanced Processing, Yamagata University, 4-3-16 Jonan, Yonezawa, Yamagata 992-8510, Japan; takashi.kurose@yz.yamagata-u.ac.jp; 3 Taisei Kogyo Co., Ltd., 26-1 Ikeda-Kitamachi, Neyagawa, Osaka 572-0073, Japan; yota_kanaya@taisei-kogyo-net.co.jp (Y.K.); shigeo_tanaka@taisei-kogyo-net.co.jp (S.T.)

**Keywords:** metal FDM, 17-4PH stainless steel, 3D printing conditions

## Abstract

17-4PH stainless steel specimens were fabricated by fused deposition of metals (FDMet) technology, which combines 17-4PH particles with an organic binder. FDMet promises a low-cost additive manufacturing process. The present research aims to clarify the influence of layer directions in the 3D printing process on the mechanical and shrinkage properties of as-sintered and as-aged specimens. All specimens (the as-sintered and as-aged specimens printed in three layer directions) exhibited high relative density (97.5–98%). The highest ultimate strengths (880 and 1140 MPa in the as-sintered and as-aged specimens, respectively) were obtained when the layer direction was perpendicular to the tensile direction. Conversely, the specimens printed with their layer direction parallel to the tensile direction presented a low ultimate strength and low strain at breakage. The fact that the specimens with their layer direction parallel to the tensile direction presented a low ultimate strength and low strain at breakage is a usual behavior of parts obtained by means of FDM. The SEM images revealed oriented binder domains in the printed parts and oriented voids in the sintered parts. It was assumed that large binder domains in the filament were oriented perpendicular to the layer directions during the fused deposition modeling printing, and remained as oriented voids after sintering. Stress concentration in the oriented void defects was likely responsible for the poor tensile properties of these specimens.

## 1. Introduction

Additive manufacturing (AM) can produce complex three-dimensional (3D) parts that are difficult to fabricate by conventional techniques. AM can also produce small lot parts at a fast speed without a mold [1,2,3,4,5]. Popular metal-AM technologies are selective laser sintering (SLS) [6,7,8,9,10,11] and electron beam melting (EBM) [12,13,14,15]. Both technologies have been widely reported and are now applied in various industries (such as biomedicine, aerospace, and military). However, these technologies are expensive to install and maintain, requiring an inert gas environment and a cooling system during operation.

Filament deposition of metals (FDMet), often called material extrusion or composite extrusion modeling, is a multi-step AM technology that promises to solve the cost problems of conventional metal AM [16,17,18,19,20,21,22,23,24,25,26,27,28]. The first step is the preparation of the feedstock, a high-filled composite of metal powder/thermoplastic organic binder. The second step forms the feedstock into filaments for the fused deposition modeling (FDM) printer. The third step prints the 3D part (the green part) through the FDM printer, which is operated at the melting temperature of the binder. The fourth step, called debinding, removes the binder from the green part. The final step (sintering) densifies the part by a thermal treatment that combines the powder particles. Two existing technologies—metal injection molding (MIM) [29,30,31] and FDM [32,33,34]—have been combined into an FDMet process. MIM is a powder metallurgical process that sinters metal particles at a temperature well below their melting point, thereby conserving heat energy. Meanwhile, FDM is a low-cost process that builds 3D parts layer by layer from the extruded filaments ejected by a hot nozzle extruder.

The FDMet process [16,17,18,19,20,21,22,23,24,25,26,27,28] has been applied to metal particles of SUS316 [20,21,22,23] and 17-4PH stainless steel (17-4PH SS) [24,25,26,27,28]. As a precipitation-hardened martensite stainless steel, 17-4PH SS is stronger and harder than SUS316. The martensite matrix of 17-4PH contains nanoscale Cu-rich spherical particles that precipitate during heat treatment (solution treatment followed by aging treatment) [35,36,37]. Accordingly, 17-4PH is used in various applications, such as aerospace, marine environments, chemical engineering, and nuclear power generation. However, the superior mechanical properties of this material reduce its formability. Near-net-shape processes such as MIM and AM are suitable processes for 17-4PH SS [6,7,8,9,11,24,25,26,27,28,29,30,31]. Wu et al. fabricated 17-4PH SS parts by FDMet, focusing on the dimensional accuracy of the sintered parts [26]. Gutierrez et al. produced 17-4PH SS dog-bone specimens and preliminarily investigated their mechanical properties and porosity [28]. Lieberwirth et al. reported the density and compression strength of cylindrical parts fabricated by FDMet [24]. Although these previous studies reported the properties of 17-4PH SS fabricated by FDMet [24,25,26,27,28], the relationship between the physical properties (i.e., mechanical properties, shrinkage by sintering) and the internal structures of the printed or sintered parts has not been investigated. Moreover, the effect of the layer directions in the FDMet processing of 17-4PH SS remains unclarified.

In the present research, the fundamental industrial properties (mechanical properties, dimensional stability, and internal structures) are related to the layer direction of 17-4PH SS parts fabricated by the FDMet process. The feedstock was composed similarly to conventional-grade feedstock in MIM, namely, 60 vol% 17-4PH SS particles and 40 vol% organic binder. Dog-bone specimens were printed in three directions: along the width, thickness, and length of the specimen. Prior to sintering, the organic binder was removed by thermal debinding. The effects of precipitation-hardening treatment on the mechanical and internal structures of the sintered 17-4PH SS parts were also investigated.

## 2. Materials and Methods

As mentioned above, the feedstock was composed of 60 vol% 17-4PH stainless steel particles and 40 vol% organic binder. The feedstock was provided by Taisei Kogyo Co., Ltd. (Osaka, Japan). The 17-4PH SS powder was water-atomized with an average particle diameter of 10 μm, and the organic binder consisted of polyoxymethylene (POM), polypropylene (PP), and paraffin wax (PW). The filament was produced by extruding the feedstock through a capillary rheometer with a 9.5 mm-diameter barrel and a 1.75 mm-diameter die (CAPILOGRAPH-1D, Toyo Seiki Seisaku-syo., Ltd., Tokyo, Japan). The extruding temperature and piston speed were 130 °C and 50 mm/min, respectively. The extruded filament (of diameter 1.73 ± 0.02 mm) was wound up below the die while cooling at room temperature.

**Figure 1:**
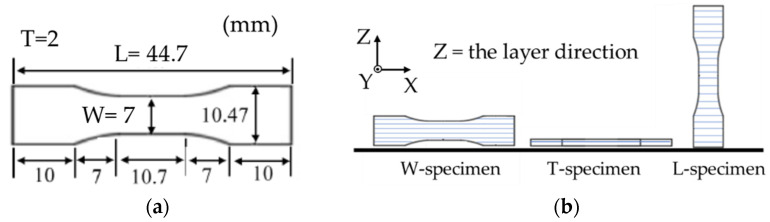
(**a**) Specimen dimensions (L = length; W = width; T = thickness) and (**b**) layer directions of the specimens with respect to the coordinate axes.

The filaments were input to a modified commercial FDM 3D printer (L-DEVO M2030TP, Fusion Technology Co., Tokyo, Japan) [23]. Because the produced filament was too brittle for printing at room temperature, a temperature-controlled chamber for the filament was prepared, and a flexible duct was connected from the chamber to the extruder unit of the 3D printer. This duct controlled the temperature of the filament. The 3D printing conditions are listed in Table 1, and the dimensions of the printed specimen are shown in Figure 1a. Here, T, W, and L denote the thickness, width, and length of the specimen, respectively. The specimens were printed in three directions of printing layers, as shown in Figure 1b, together with the coordinate axes. The extruder unit with the nozzle moved in the X and Y directions, and the printing stage moved only along the Z direction (defined as the layer direction). The printing patterns of each layer were as follows. First, two outside lines were printed as the “outer wall.” The inside was then filled at a raster angle of 45°/−45°. All layers were printed at the nominal infill density (100%) with the rectilinear infill pattern. The 3D printed parts were called the green parts.

The sintered part was fabricated by continuous thermal debinding and sintering in the same vacuum furnace (Shimazu Industrial Systems Co., Ltd., Otsu, Shiga, Japan). First, the green part was de-bound at 600 °C for 2 h under a nitrogen gas atmosphere. Subsequently, the atmosphere was changed to argon gas, and the part was sintered at 1280 °C for 2 h. The sintered parts, called the as-sintered specimens, were subjected to the sequential solution and aging treatments. When 17-4PH is subjected to a solution heat treatment between 1020 and 1060 °C, and it is aged at a predetermined temperature, a Cu-rich phase is precipitated inside of the material and they increase the strength and hardness of the 17-4PH. Therefore, the solution treatment was performed at 1040 °C for 1 h followed by argon gas quenching. The aging treatment was performed at approximately 480 °C for 1 h (condition H900, peak-aging). The treated parts were called as-aged specimens. Regardless of their layer direction, all specimens were processed in the same position, namely, with the thickness direction of the specimen parallel to the direction of gravity on a flat plate in the furnace.

The relative densities of the as-sintered and as-aged specimens were calculated from the theoretical density of 17-4PH (7.78 g/cm^3^) and the experimental density. The experimental relative densities of the as-sintered and as-aged specimens were estimated by the Archimedes method, which uses the weight measurements in water and air. The dimensional linear shrinkages from the green part to the as-sintered and as-aged specimens were estimated by measuring the dimensions of each part with a caliper. The tensile test was performed in a universal testing machine with a 100-kN load cell (Autograph AG-10TD, Shimazu Corp., Kyoto, Japan). The crosshead speed was 2 mm/min. Three specimens were tested under each condition to ensure repeatability.

The internal structures of the feedstock, filaments, green part, as-sintered, and as-aged specimens were analyzed. The fracture surfaces of the feedstock, filaments, and green parts were examined at room temperature. The cross-sections of the as-sintered and as-aged specimens were polished with a polishing machine (ML-150P, Maruto Instrument, Co., Ltd., Tokyo, Japan). The cross-sectional surfaces perpendicular to the length and thickness directions of the as-sintered and as-aged specimens were electrolytically etched to reveal their macro-internal structures. The etching was performed in an aqueous solution of 10% oxalic acid with a current density of 1.0 A/cm^2^ at room temperature. The fractured surfaces of the as-sintered and as-aged specimens after the tensile test were also observed. These observations were performed using scanning electron microscopy (SEM) (TM3030 plus, Hitachi High-Technologies Corp., Tokyo, Japan) and energy-dispersive X-ray spectroscopy (EDX) analysis. The microstructures of the as-sintered and as-aged T-specimens were observed after the chemical etching of their polishing surfaces. The chemical treatment was performed in a solution of HCl (5.0 g), picric acid (1.0 g), and ethanol (100.0 g) at room temperature. The chemically etched surfaces were observed under a digital microscope (VHX-950F, KEYENCE Corp., Osaka, Japan).

## 3. Results and Discussion

### 3.1. Internal Structure

**Figure 2:**
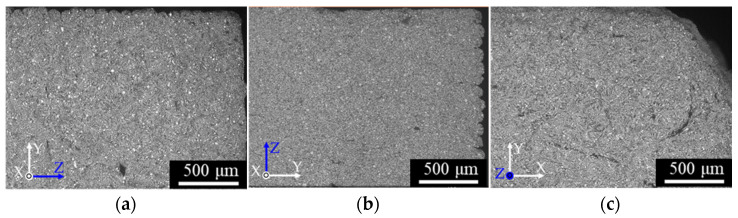
SEM micrographs of the cross-sections perpendicular to the length direction of the green specimens: (**a**) W-specimen, (**b**) T-specimen, (**c**) L-specimen.

**Figure 3:**
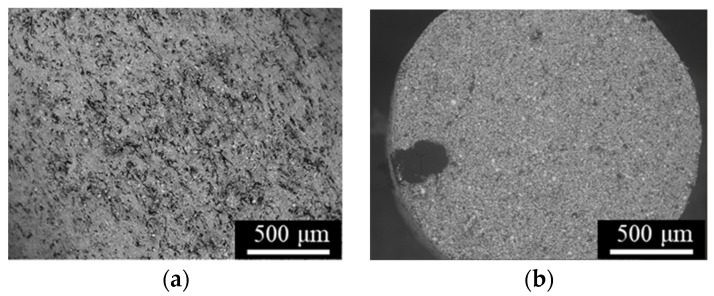
SEM micrographs of the fracture surfaces of (**a**) feedstock and (**b**) a filament.

Figure 2 shows the cross-sections perpendicular to the length of the green specimens printed in various layer directions. After SEM analysis with EDX, the dark spots in the images were confirmed as binder components rather than voids. The inside of the green specimens was judged to be fully filled with the material. Panels (a) and (b) of Figure 3 show the fracture surfaces of the feedstock and filaments, respectively. Dark spots were observed in both images. These spots, sized several tens of µm in the feedstock and several hundreds of µm in the filament, were assumed as PP or POM, which are immiscible to PW and have higher melting and crystallization temperatures than PW. The formation temperature of the filament (130 °C) was below the melting points of the POM and PP binder components. Moreover, the filament was produced through simple piston-type extrusion, which generates no strong mixing force in the material. Therefore, the small segregates in the feedstock were aggregated into larger structures during the filament processing. These large aggregates remained after 3D printing and were observed as binder domains in Figure 2.

**Figure 4:**
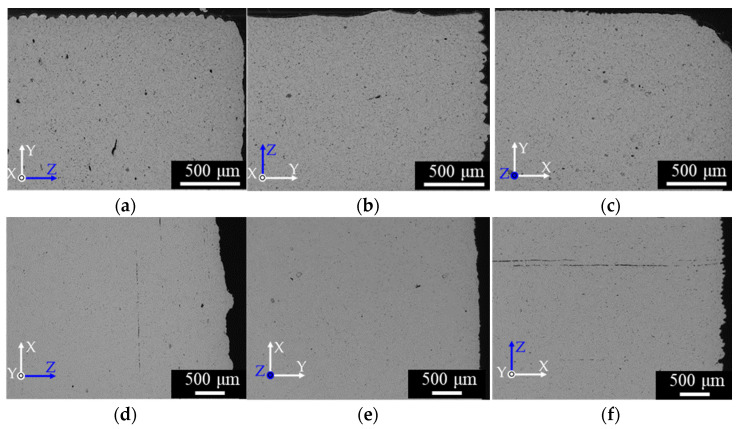
SEM micrographs of cross-sections of the as-sintered specimens observed perpendicular to the lengths of the (**a**) W-specimen, (**b**) T-specimen, and (**c**) L-specimen, and perpendicular to the thicknesses of the (**d**) W-specimen, (**e**) T-specimen, and (**f**) L-specimen.

Figure 4 shows the cross-sections perpendicular to the length (a)–(c) and thickness directions (d)–(f) of the as-sintered specimens. Many voids appeared in all specimens. In panels (d) and (f), the voids were highly oriented perpendicular to the layer direction Z. This observation is attributable to the orientation of the binder domains during the 3D printing process. The nozzle temperature of the FDM printing was 170 °C, above the melting points of the binder components, and the nozzle diameter was 400 µm across with a layer height of 100 µm in the Z direction. Therefore, the extruded material was deformed by shearing between the moving nozzle and the solidified underlayer; consequently, the melted binder domain was oriented parallel to the nozzle-moving direction (perpendicular to the layer direction Z). The voids formed by the debinding of large organic binder domains were too large to be buried by sintering, so remained in the as-sintered specimens. The lack of oriented voids in the XY plane in Figure 4e can be explained by the slit-like shapes of the voids oriented in that plane.

**Figure 5:**
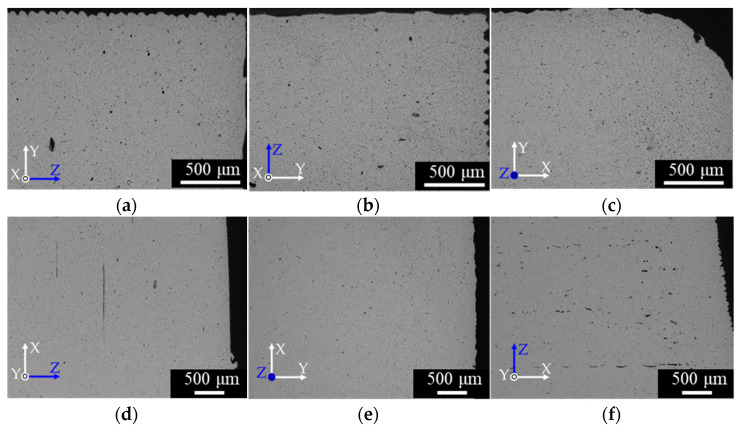
SEM micrographs of cross-sections of the as-aged specimens perpendicular to the lengths of the (**a**) W-specimen, (**b**) T-specimen, and (**c**) L-specimen, and perpendicular to the thicknesses of the (**d**) W-specimen (**e**) T-specimen and (**f**) L-specimen.

**Figure 6:**
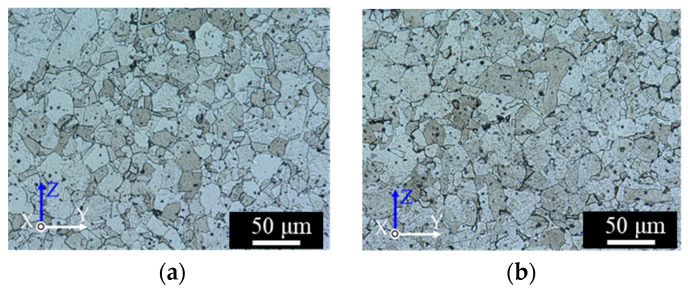
Digital-microscope micrographs of the cross-sections perpendicular to the length of the T-specimens: (**a**) as-sintered, and (**b**) as-aged.

Cross-sections of the as-aged specimens are shown in Figure 5. The structure patterns were similar to those of the as-sintered specimens, suggesting that the solution and aging treatments little affected the macroscopic structure. The oriented voids in Figure 4 and Figure 5 can be fatal defects that significantly impact the tensile properties of the specimens (especially those of the L-specimens, in which the voids are oriented perpendicular to the tensile direction). Panels (a) and (b) of Figure 6 show the microstructures of the as-sintered and as-aged T samples, respectively, perpendicular to the length direction. The dark and bright grains are the martensite and ferrite phases, respectively. The spherical particles with sizes of several µm were identified as SiO_2_ inclusions (EDX). SiO_2_ inclusions have also been confirmed in previous reports on 17-4PH MIM [29,31]. The as-aged specimens exhibited more dark grains than the as-sintered samples. Specimens fabricated by the SLS process present geometric patterns caused by melt bonding under laser heating [6,7,8,9,11], however, those layer-boundary patterns were not observed in the as-sintered and as-aged specimens of the present study.

### 3.2. Relative Density

**Figure 7:**
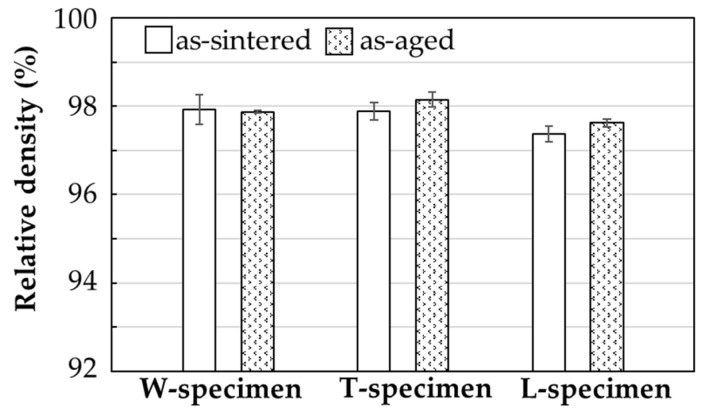
Relative densities of the as-sintered and as-aged specimens printed in various layer directions.

The relative densities of the as-sintered and as-aged specimens are shown in Figure 7. The relative densities were high and similar along all layer directions. Among the as-sintered and as-aged samples, the highest relative densities were 97.9% (W-specimen) and 98.1% (T-specimen), respectively. Wu et al. produced a small 17-4PH SS part by the FDMet process and reported a relative density of 91% [25]. Gutierrez et al. fabricated 17-4PH SS dog-bone specimens with an average porosity of 4.3% (relative density = 95.7%) [28]. The values obtained in the present study meet the standard values (density > 7.5 g/cm^3^; relative density > 96.2%) for sintered-metal injection-molded materials issued by the Japan Powder Metallurgy Association (JPMA S01:2014) [38]. The high relative density can be explained by the full packing of the material inside the green specimens, and the small volume of the large organic binder domains in the green parts and filaments, which remain as voids in the as-sintered and as-aged specimens.

### 3.3. Tensile Property

**Figure 8:**
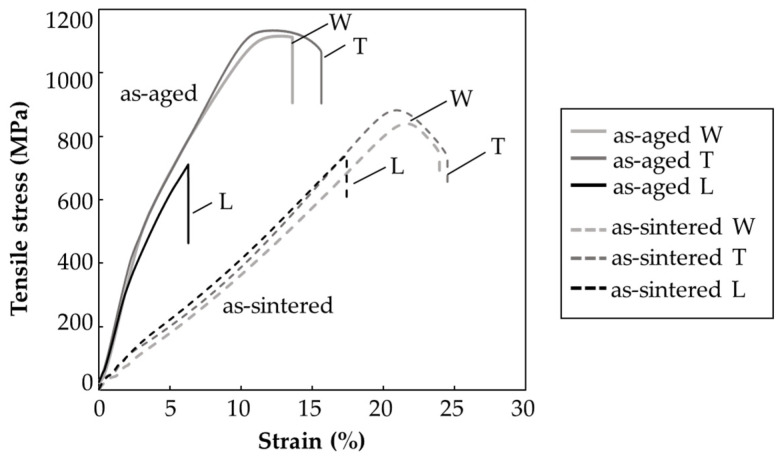
Tensile stress–strain curves of the as-sintered and as-aged specimens printed in various layer directions.

**Figure 9:**
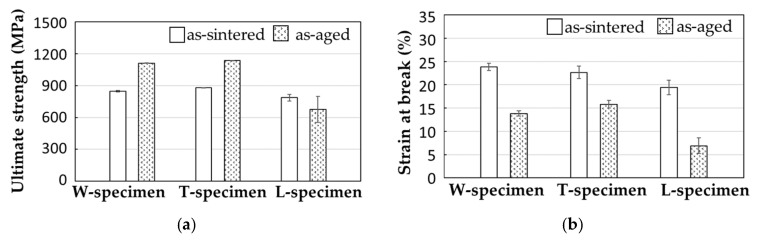
Results of the tensile test: (**a**) ultimate strength and (**b**) strain at break of the as-sintered and as-aged specimens printed in various layer directions.

Figure 8 shows the tensile stress–strain curves of the as-sintered and as-aged specimens, and Figure 9 summarizes their average ultimate strength and strain at break. The as-sintered W- and T-specimens exhibited strengths of 840 and 880 MPa, respectively, and strains at break of 24% and 23%, respectively. These values meet the JMPA standards (tensile strength > 800 MPa, strain at break > 4%; JPMA S01:2014) [39]. The strength and strain at break were lower in the L-specimen (780 MPa and < 20%, respectively). After the solution and aging treatment, the strengths of the W- and T-specimens improved to approximately 1100 and 1140 MPa, respectively. The strain at break of the W- and T-specimens was lower in the as-aged specimens (~13%) than in the as-sintered specimens (~16%). Although the strains met the JPMA standards (> 2%, JPMA S01:2014), the strengths were slightly below the standards (tensile strength > 1200 MPa, JPMA S01:2014) [39]. In general, the solution and aging treatment strengthened the tensile strength of 17-4PH SS by forming nanoscale Cu-rich spherical precipitates in the martensite matrix, which prevents dislocation movements. Consequently, the deformation resistance was improved [6,7,8,11,29,35,36,37]. However, the strength of the L-specimen was not improved by the H900 heat treatment; in fact, it was slightly decreased from that of the as-sintered specimens.

**Figure 10:**
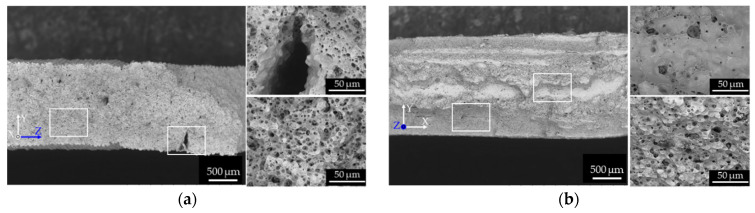

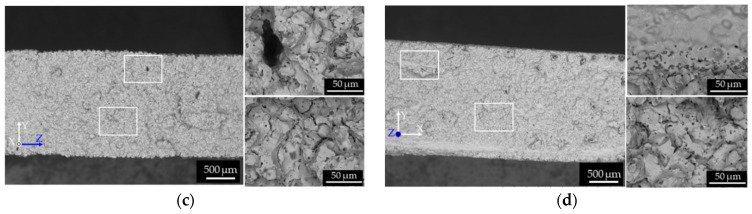
SEM micrographs of the fracture surface of the (**a**) as-sintered W-specimen, (**b**) as-sintered L-specimen, (**c**) as-aged W-specimen, and (**d**) as-aged L-specimen. The right sub-panels show two higher-magnification micrographs of the areas enclosed by the rectangular frames on the fracture surfaces (left sub-panel).

To investigate why the tensile properties were lower in the L-specimens than in the W- and T- specimens, the fracture surfaces of the as-sintered and as-aged specimens after the tensile tests were observed by SEM (Figure 10). Dimple patterns that typify ductile fracture patterns appeared across the entire fracture surface of the as-sintered W-specimens (Figure 10a). Similar fracture patterns were observed in the as-sintered T-specimens. In contrast, the dimple-fracture structure of the as-sintered L-specimen was interspersed with smooth areas (Figure 10b). Quasi-cleavage brittle fracture patterns were observed over the entire surface of the as-aged W-specimens (Figure 10c), and similar patterns appeared on the as-aged T-specimens. These results indicate that the as-aged W- and T-specimens failed in a more brittle manner, with more limited plastic deformation than the as-sintered specimens. Meanwhile, the quasi-cleavage brittle fracture surface of the as-aged L-specimens was interspersed with smooth areas (Figure 10d). These smooth areas were likely caused by the initially oriented voids in the specimens, as demonstrated in Figure 4f and Figure 5f.

**Figure 11:**
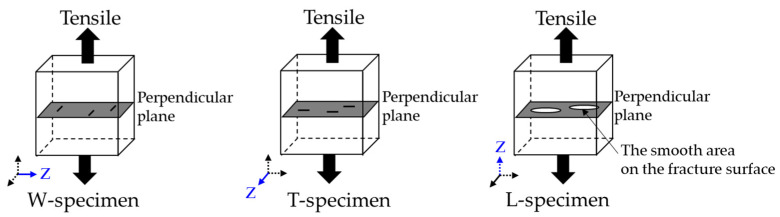
Possible mechanisms of anisotropic tensile properties in different layer directions.

Clearly, the voids in all specimens were oriented perpendicularly to the layer direction. Figure 11 displays the relationship between the oriented voids and tensile direction of the specimens in each layer direction. The voids were oriented parallel to the tensile direction in the W- and T-specimens, but perpendicular to the tensile direction in the L-specimens. The voids in the L-specimens, where the stress was concentrated, were sensitive crack sites and the origin of fracture. Therefore, they decreased the ultimate strength and strain at break. Susan et al. [39] investigated the defect susceptibility of cast 17-4PH SS parts having internal defects (casting porosity) after various aging treatments (H900, H1025, H1100). According to their report, the high-strength treatment made the parts more brittle in the same area-fraction porosity of their fracture surfaces, and hence degraded their strain at break. Brittle materials are susceptible to fracture and characterized by decreased strain at break, we concluded that the higher defects susceptivity of as-aged L-specimens reduced the mechanical properties of it from those of the as-sintered L-specimens.

### 3.4. Dimensional Linear Shrinkage

**Figure 12:**
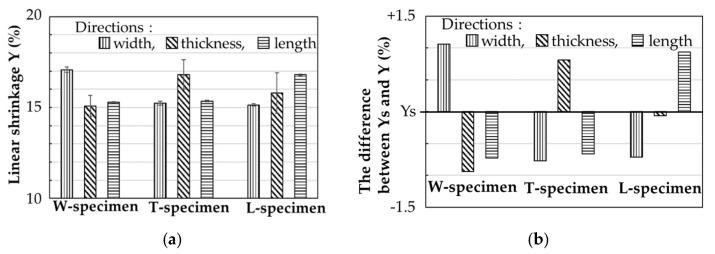
(**a**) Measured linear shrinkage Y, and (**b**) differences between the measured Y and theoretical linear shrinkage Ys of the as-sintered specimens printed in various layer directions.

Figure 12 shows the measured linear shrinkage Y from the green part to the as-sintered part for specimens printed in different layer directions. The difference between Y and the theoretical linear shrinkage $Y_{S}$ are also shown. Here, the theoretical linear shrinkage $Y_{S}$ is calculated as

(1) $$Y_{S}=1-\;{\left\{\varphi /\left(\rho /\rho_{t}\right)\right\}}^{1/3}$$

where φ is the solid feedstock loading, ρ is the final density of the as-sintered specimen, and $\rho_{t}$ is the theoretical density of the material [30]. As confirmed in Figure 12, the shrinkage behavior was anisotropic in all specimens, and it was always higher in the layer direction than in other directions. For example, the W-specimens were more shrunken in the width direction (i.e., its layer direction) than in the length and thickness directions. All specimens were sintered in the same condition with their thickness dimension oriented in the direction of gravity. Table 2 shows the dimensional shrinkage values of the as-sintered and as-aged specimens. Anisotropic shrinkage after sintering has been reported in previous studies of FDMet-fabricated parts [23,24,25]. The binder domains were oriented perpendicular to the layer direction and were both thin and thick in the green parts. The thin voids formed by debinding of the thin binder domain were buried by sintering of the metal particles, causing higher linear shrinkage in the layer direction than in other directions. Meanwhile, the thick binder domain retained its visible voids oriented perpendicular to the layer direction (Figure 4d,f). The thick binder domains are unlikely to contribute to the shrinkage of the specimen because the metal particles were not sintered across thick voids originated from the binder domain. This mechanism would explain the higher shrinkage in the layer direction of all specimens than in the other directions.

## 4. Conclusions

In the research, 17-4PH SS specimens were successfully fabricated from the filaments consisting of 17-4PH SS powder and organic binder through the FDMet process. The as-sintered and as-aged specimens, regardless of printing layer direction, achieved high relative density (97.5–98%).

Internal SEM observations revealed oriented voids perpendicular to the layer direction in the as-sintered and as-aged specimens, implying that the binder aggregates in the filament became the binder domain and were oriented during the 3D print process. The voids formed by the debinding of large organic binder domains, which were too large to be buried by sintering, remained as voids even in the as-sintered and as-aged specimens.

Among the as-sintered specimens, the specimen printed with its layer direction perpendicular to the tensile direction delivered the highest ultimate strength (880 MPa, improving to 1140 MPa after solution and aging treatment). The strains at break of the as-aged W- and T-specimens were decreased by the solution and aging treatment. Meanwhile, the ultimate strength and strain at break of the L-specimen, printed with its layer direction parallel to the tensile direction, were deficient. The anisotropic mechanical properties can be explained by the presence of oriented voids perpendicular to the tensile direction. These voids concentrated the stress and initiated fractures.

The linear shrinkage was always higher in the layer direction than in other directions. This anisotropy was observed in both heat-treated and non-heat-treated specimens printed in different layer directions and was explained by an oriented thin binder domain developed perpendicularly to the layer direction in the green parts. The thin voids, formed by debinding the thin domain, were buried by the sintering of metal particles, enhancing the linear shrinkage in the layer direction.

The oriented binder domain was concluded to be the cause of the mechanical and dimensional anisotropy, it is supposed to reduce the anisotropy by eliminating or reducing the binder aggregation through improvements in material and process technologies. It is expected the aggregation of binder can be avoided if the polymer composition is optimized for FDMet since the binder deployed in this study was developed for MIM manufacturing.

Mechanical properties of the metal part fabricated with the FDMet technique have the potential to meet the industrial standards for sintered-metal-injection-molded material. Although this process can be a low-cost AM process for thin and small-sized parts, the anisotropic shrinkage and mechanical properties have to be improved by research and development of material and process and should be also considered in design under product development for commercialization.

## Figures and Tables

**Table 1:** 3D printing conditions.

Printing Parameter	Value	Unit	Printing Parameter	Value	Unit
Nozzle diameter	0.4	mm	Printing rate	10	mm/min
Nozzle temperature	170	°C	Layer height	0.1	mm
Chamber temperature	80	°C	Infill ratio	100	%
Stage temperature	70	°C	Outer shell thickness	0.8	mm

**Table 2:** Measured dimensional shrinkages from the green parts to as-sintered/as-aged samples printed in the W, T, and L layer directions.

	Width (%) As-Sintered/As-Aged	Thickness (%) As-Sintered/As-Aged	Length (%) As-Sintered/As-Aged
W	17.1/16.8	15.1/14.6	15.3/15.3
T	15.2/15.3	16.8/16.5	15.3/15.4
L	15.1/14.7	15.8/15.2	16.8/16.9

## Data Availability

Data sharing is not applicable to this article.
